# Liposome-Mediated Bacterial Ferroptosis-like Death: A Novel Paradigm for Antimicrobial Therapy

**DOI:** 10.3390/antibiotics15080738

**Published:** 2026-07-30

**Authors:** Rui Yang, Zhengwei Huang, Xuejuan Zhang

**Affiliations:** 1 State Key Laboratory of Bioactive Molecules and Druggability Assessment, Guangdong Basic Research Center of Excellence for Natural Bioactive Molecules and Discovery of Innovative Drugs, College of Pharmacy, Jinan University, Guangzhou 511443, China; ruiyang@stu2023.jnu.edu.cn; 2 Key Laboratory of Drug-Targeting and Drug Delivery System of the Education Ministry, West China School of Pharmacy, Sichuan University, Chengdu 610041, China

**Keywords:** bacterial infection, antibacterial, ferroptosis-like death, nanomedicine, liposome

## Abstract

The growing global crisis of antimicrobial resistance (AMR) urgently demands non-classical therapies capable of evading established resistance mechanisms. Bacterial ferroptosis-like death, an iron-dependent process driven by lipid peroxidation, offers a promising strategy to circumvent conventional drug resistance. However, the clinical translation of ferroptosis-like inducers is hindered by poor water solubility and off-target systemic toxicity. Featuring tunable physicochemical characteristics and proven clinical biosafety, liposomes stand out as a viable platform to resolve these translational bottlenecks. Although antibacterial nanomedicines have been extensively investigated, the specific synergies between liposomal engineering and bacterial ferroptosis-like pathways remain underexplored. To bridge this gap, this review systematically delineates the molecular cascades of bacterial ferroptosis-like death and highlights unique mechanistic advantages of liposomes. Importantly, liposome-mediated ferroptosis-like antibacterial therapy faces prominent translational challenges, including biosafety concerns, insufficient stability, and targeting limitations. This review further outlines advanced liposomal engineering strategies to tackle the above obstacles and discusses pressing questions that should be the focus of future ferroptosis-like research. By integrating multidisciplinary research outcomes, this review may provide insights and feasible design guidelines to advance the translational development of liposomal ferroptosis-like inducers against AMR infections.

## 1. Introduction

The vast majority of bacteria, both pathogenic and saprophytic, are unicellular organisms that reproduce by binary fission [1]. Bacterial infections occur when bacteria enter the body, multiply in number, and trigger a reaction in the body. These infections manifest through a remarkably broad spectrum of pathologies, including cutaneous cellulitis caused by *Staphylococcus aureus* [2], life-threatening bacterial pneumonia resulting from pulmonary infection with *Streptococcus pneumoniae* [3], and gastrointestinal disorders such as gastritis and gastric ulcers attributed to *Helicobacter pylori* [4], against which the stomach’s acidic environment offers no total protection. Bacterial infections have become a globally escalating health problem, claiming an estimated 7.7 million deaths worldwide annually attributed to these infections in 2019 and making their position as the world’s second leading cause of death [5]. It is evident that bacterial infections are a major public health problem that seriously threaten human health.

Bacterial infections play a dual role in clinical medicine: they can not only arise as independent entities, affecting otherwise healthy individuals, but also as opportunistic burdens that superimpose upon chronic pathologies; hence exacerbating the underlying pathology. For instance, diabetic patients are highly susceptible to bacterial infections, particularly in wounds, often accompanied by diabetic foot ulcers, which is one of the main causes of amputation in patients [6]. In addition, this pattern of vulnerability is observed in patients with liver cirrhosis, whose compromised immune defenses render them significantly more susceptible than the general population. Clinical data indicates that 25% to 50% of hospitalized cirrhotic patients will develop bacterial complications [7]. The above indicates that bacterial infection is also an aggravating factor that cannot be ignored in the chronic course of the disease, and more proactive prevention and intervention strategies are urgently needed.

In recent years, the emergence of “superbugs”, most notably Methicillin-resistant *Staphylococcus aureus* (MRSA) even has caused diseases like sepsis, endocarditis, pneumonia, skin and soft tissue infections, bone and joint infections, etc. [8]. Therefore, the clinical situation of bacterial infections ranging from high global mortality rates to the relentless rise in superbugs is not optimistic. From this standpoint, it can be seen that the clinical situation of treating bacterial infections has become increasingly serious and tense. The entire world attaches great importance to the research and development of antibacterial drugs and their pharmaceutical formulations.

In this context, we propose bacterial ferroptosis-like death nanomedicine as candidate antimicrobial formulations to provide alternative strategies for addressing severe drug resistance in bacterial infections. This review will discuss the following aspects: the clinical status of bacterial infections and antimicrobial resistance issues, existing strategies to combat resistance and their limitations, the unique advantages of bacterial ferroptosis-like death as a novel antimicrobial mechanism, physicochemical limitations of small-molecule bacterial ferroptosis-like death inducers, construction strategies of liposomal delivery systems and their clinical application prospects. We hope this review article can provide theoretical foundations and development insights for designing a novel antimicrobial nanomedicine that circumvents conventional resistance mechanisms, and then facilitates the clinical translation of bacterial ferroptosis-like death-inducing nanoformulations.

## 2. Method

Document retrieving was performed at 12 a.m., 3 May 2026 (UST + 8 time zone). The documents of interest were retrieved from the Science Citation Index-Expanded collection in the Web of Science Core Collection, with the search set of “topic = ferroptosis”, “topic = liposomes.” AND “topic = ferroptosis-like” The type of documents was restricted to “article” and “review” and the publication time 2020~2026. The duplicates were precluded. The retrieved documents were exported in the form of “full record and cited references” as plain text or tab delimited (Win, UTF-8) files.

## 3. Clinical Therapies for Bacterial Infections and Existing Problems

### 3.1. Drug Therapy Is the Core Measures

Despite the continuous development of adjuvant therapies for bacterial infections (such as surgical drainage), antibacterial treatment remains the cornerstone and preferred approach for some bacterial diseases at present [2]. Traditional antibiotics have historically achieved remarkable success in controlling bacterial infections and saving countless lives, establishing their indispensable role in modern medicine. The World Health Organization (WHO) guidelines clearly list drug therapy as the core intervention for bacterial infections [9]. The most prevalent bacterial infectious diseases, along with their established therapeutic regimens and mechanisms, are summarized and presented in Table 1**.**

### 3.2. The Increase in Antimicrobial Resistance

Although the clinical application of these agents is frequently associated with adverse reactions ranging from hypersensitivity and gastrointestinal disturbances to severe organ-specific toxicities such as hepatotoxicity, nephrotoxicity, and neurotoxicity, the most critical challenge currently facing the field is the increase in antimicrobial resistance (AMR); it occurs when bacteria, fungi, viruses, and parasites evolve over time, acquiring new mechanisms to evade antimicrobial treatment [25]. Currently, AMR is a rapidly growing global phenomenon, emerging as one of the top ten global threats declared by WHO [25]. WHO has forewarned that drug-resistant infections could lead to 10 million deaths annually by 2050 if no new treatment strategies are developed [26].

### 3.3. The Mechanisms of Bacterial Resistance

**Figure 1:**
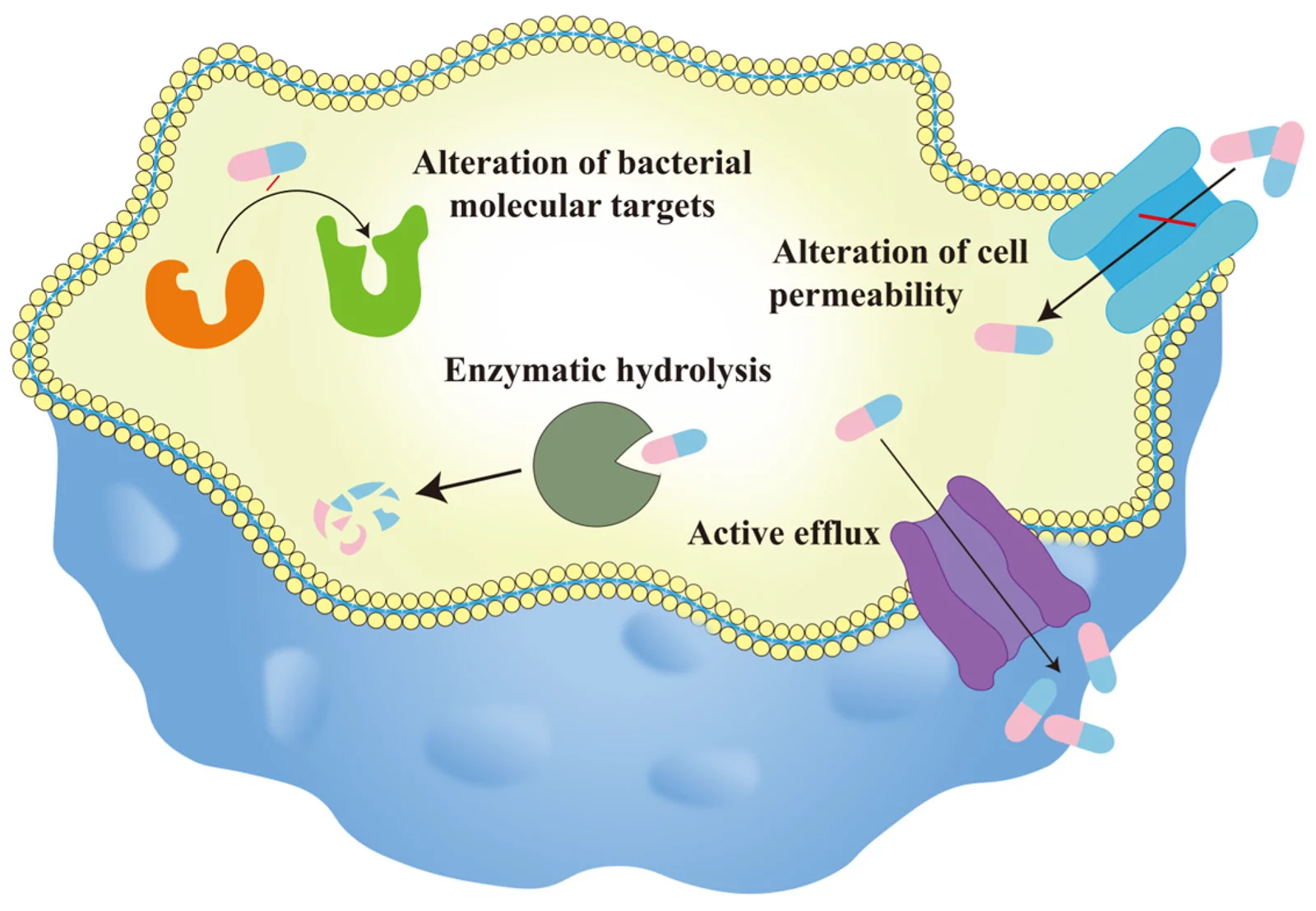
Mechanisms of bacterial resistance.

Addressing this critical challenge requires a deeper understanding of the underlying resistance mechanisms, and it is essential to categorize the diverse strategies bacteria employ to develop resistance. The major four mechanisms of bacterial drug resistance are illustrated in Figure 1.

#### 3.3.1. Enzymatic Hydrolysis

Enzyme-catalyzed hydrolysis is extensively employed by bacteria to destroy the reactive drug conjugates produced by either pathogenic drug-resistant strains or antibiotic-producing bacteria. The mechanisms of action of these enzymes include impairing the enzyme’s active site and preventing binding [27]. A representative example is β-lactamase, a hydrolase capable of catalyzing the cleavage of the amide bond (-CO-NH structure) and opening the β-lactam ring of antibiotics, which leads to the degradation of β-lactams [28].

#### 3.3.2. Active Efflux

On the bacterial cell membrane, there are proteins known as “efflux pumps”. These proteins regulate the flow of toxic substances from the intracellular environment of the cell to the extracellular environment [29]. Bacterial efflux pumps are the most rapid and efficient resistance mechanism for bacteria to respond to stress, and they are present in nearly all bacteria. Although efflux pumps enhance the survival ability of microorganisms under harsh conditions, they also increase the microorganisms’ resistance to antibiotics and antibacterial agents [30].

#### 3.3.3. Alteration of Bacterial Molecular Target

A critical strategy for bacterial resistance acquisition is the alteration of drug target structures, most notably exemplified by ribosome modification via ribosomal ribonucleic acid (rRNA) methylation. This methylation targets key nucleotides within the antibiotic binding site, inducing steric hindrance that impairs the binding affinity of the drug. Given that the binding sites of various antibiotics partially overlap, target modification at a single nucleotide can confer resistance to multiple classes of antibiotics [31,32]. In this way, a microscopic alteration at the binding site can translate into a macroscopic resistance phenotype.

#### 3.3.4. Alteration of Cell Permeability

Reducing cell envelope permeability is a fundamental resistance mechanism, achieved primarily through membrane modifications and biofilm formation. Biofilms are complex matrices rich in proteins, lipids, and polysaccharides [33] that adhere to surfaces and encapsulate bacterial cells, forming structured microbial aggregates. This structure not only impedes the penetration of antimicrobial agents but also adsorbs inactivating enzymes capable of hydrolyzing such drugs. Compared with planktonic bacteria, the drug resistance, especially antibiotic resistance, of bacteria residing in biofilms increases by 10- to 1000-fold [34].

In summary, antimicrobial resistance has escalated into a global public health crisis. Diverse resistance mechanisms often operate synergistically to erode drug efficacy and drive therapeutic failure. Therefore, relying solely on the incremental development of conventional antibiotics is insufficient, highlighting an urgent need for novel antibacterial strategies.

## 4. Current Strategies to Overcome Drug Resistance

**Figure 2:**
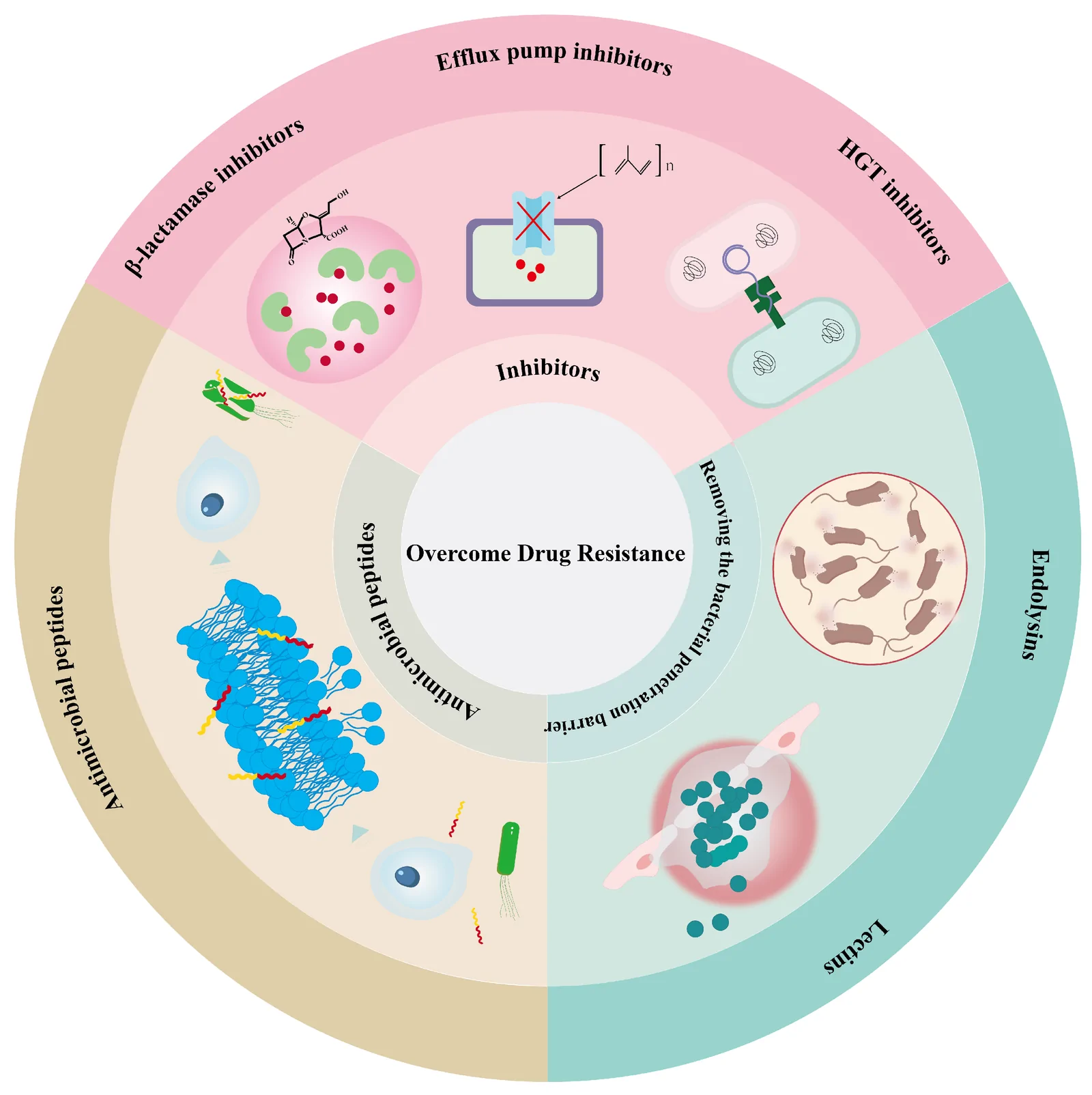
Current methods to overcome drug resistance of antibiotics.

In view of the mechanisms outlined above, a range of promising strategies have emerged, aiming to either overcome existing resistance or disarm pathogens, as illustrated in Figure 2.

### 4.1. Inhibitors

Inhibitors have gained wide use in recent years to delay the emergence of bacterial resistance due to their high safety, low cost, and high efficacy [30]. For instance, β-lactamase inhibitors bind to the active site of β-lactamase, thus protecting antibiotics from enzymatic hydrolysis [28]. Efflux pump inhibitors (EPIs) restore intracellular antibiotic accumulation and minimize the emergence of resistant mutants [35] by saturating efflux capacity [36], disrupting active transport energy (e.g., though proton motive force (PMF) dissipation) [37], or inactivate the outward transport of drugs [30].

### 4.2. Removing the Bacterial Penetration Barrier

Phage-derived endolysins serve as potent antimicrobials by degrading peptidoglycan [38], the core cell wall component, thus triggering inside-out bacterial lysis [39]. Bacterial biofilms are complex three-dimensional aggregates composed of bacteria and extracellular polysaccharides (EPS) [34]. Lectins can disrupt bacterial adhesion, biofilm formation, and invasion of host cells, making them a promising approach to overcoming biofilms [40].

### 4.3. Antimicrobial Peptides (AMPs)

AMPs, generally containing fewer than 50 amino acids, destabilize bacteria by electrostatically targeting cell membranes and perturbing intracellular processes. This dual-action mechanism provides broad-spectrum efficacy against Gram-positive and Gram-negative bacteria while conferring a low propensity for resistance development [41]. In addition, certain AMPs are capable of penetrating the bacterial membrane to enter the cytoplasm, binding to ribosomes and inhibiting protein translation [42]. This multi-target approach positions AMPs as highly promising alternatives to conventional antibiotics [43].

However, the clinical translation of these strategies is hampered by safety, pharmacokinetic, and efficacy limitations. Safety-wise, EPIs and AMPs risk off-target cytotoxicity to host cells [44,45], while exogenous endolysins may provoke neutralizing immune responses [46]. Pharmacokinetically, proteinaceous agents (AMPs and endolysins) suffer from rapid proteolytic degradation and short in vivo half-lives [47,48]. Additionally, co-administered enzyme inhibitors often exhibit mismatched tissue distribution with main antibiotics, reducing localized efficacy [49]. Finally, target coverage remains narrow: EPIs and β-lactamase inhibitors frequently fail against diverse subtypes or extensively drug-resistant strains [50,51], whereas endolysins are blocked by Gram-negative outer membranes and peptidoglycan modifications [52]. Therefore, it is urgent to seek new strategies avoiding the above shortages to surmount bacterial drug resistance.

## 5. Bacterial Ferroptosis-like Death as a Feasible Alternative Strategy

### 5.1. The Mechanisms of Ferroptosis in Eukaryotic Cells

In 2012, Dixon et al. first reported ferroptosis [53], which is a novel form of cell death triggered by the accumulation of iron-dependent lipid peroxidation. This form of cell death is distinct from traditional forms like apoptosis and necrosis, the mechanisms of ferroptosis include the following points:

#### 5.1.1. Dysregulation of the Cellular Antioxidant Defense System

Glutathione peroxidase 4 (GPX4) pathway: Cells import extracellular cystine via the system Xc- transporter (a cystine/glutamate antiporter), and reduced them to cysteine to synthesize glutathione (GSH). GPX4 then utilizes GSH as a vital cofactor to catalyze the reduction in toxic lipid hydroperoxides (L-OOH) into non-toxic lipid alcohols (L-OH). Consequently, if system Xc- is compromised or GPX4 activity is inhibited, the cell loses its capacity to eliminate lipid peroxides, hence triggering ferroptosis [54].

Ferroptosis suppressor protein 1 (FSP1) pathway: FSP1 uses nicotinamide adenine dinucleotide phosphate (NADPH) to coenzyme Q_10_ (CoQ_10_) to ubiquinol (CoQ_10_H_2_), acting as a lipid-soluble free radical scavenging antioxidant, directly terminating the lipid peroxidation chain reaction. Inhibiting FSP1 can enhance ferroptosis sensitivity [55,56].

#### 5.1.2. Iron Accumulation and Lipid Peroxidation

The hallmark of ferroptosis is iron accumulation and unrestrained lipid peroxidation, and these are also the main driving factors for the occurrence of ferroptosis [54]. Fe^2+^ in the cytoplasm forms various iron-binding complexes. When the iron-binding complexes reach saturation, the excess Fe^2+^ will accumulate in the labile iron pool (LIP). Subsequently, it directly catalyzes the decomposition of H_2_O_2_ into hydroxyl radicals through the Fenton reaction and promotes the accumulation of intracellular reactive oxygen species (ROS) and lipid peroxidation. The content of polyunsaturated fatty acids (PUFAs), such as arachidonic acid (AA) and adrenic acid (AdA), is relatively high in cells and organelles. This makes them susceptible to lipid peroxidation, leading to the generation of lipid peroxides. If not neutralized by cellular defense mechanisms, it will lead to damage to cell integrity and function [57,58].

#### 5.1.3. Others

Dihydroorotate dehydrogenase (DHODH), a mitochondrial enzyme that mitigates ferroptosis by reducing CoQ_10_ to CoQ_10_H_2_ [59]. Thus, DHODH inhibition can promote mitochondrial lipid peroxidation and ferroptosis [60].

From the above discussion, it is evident that the process of ferroptosis results from the synergistic interplay between the collapse of cellular antioxidant surveillance systems, primarily the GPX4 and FSP1 pathways, and the escalation of iron-catalyzed oxidative stress. Ferroptosis has been previously demonstrated to play a key role in inhibiting the growth of tumor cells [61,62]. Relevant studies have verified that ferroptosis can induce immunogenic cell death and trigger effective anti-tumor immune responses [63]. These findings indicate that ferroptosis is a promising strategy for effective cell killing. It should be noticed that bacteria are also “cells”, and thus cell killing effects may be promising for bacterial infections.

### 5.2. The Mechanism of Bacterial Ferroptosis-like Death

Distinct from eukaryotic host cells and tumor cells, which rely on the GPX4/GSH axis to modulate ferroptosis, or immune cells wherein lipid peroxidation dictates immunoregulatory phenotypes, bacteria lack mitochondria and other eukaryotic organelles; nevertheless, they possess Fe-S cluster assembly systems and lipid metabolic pathways that resemble those involved in eukaryotic ferroptosis. The disruption of these systems by iron overload or oxidative imbalance can cause bacterial ferroptosis-like death, leading to the destruction of bacterial membranes and the inhibition of basic metabolic functions [64]. Therefore, typical mammalian ferroptosis relies on regulatory pathways involving GPX4, FSP1, and DHODH. In contrast, simple iron-mediated oxidative killing relies merely on non-specific Fenton reactions to cause cellular damage [53]. However, the specific mechanisms underlying bacterial ferroptosis-like death are primarily driven by the following three aspects:

#### 5.2.1. Intracellular Iron Overload

Exogenous ions (e.g., Fe^2+^, Fe^3+^) upregulate bacterial iron uptake pathways. The accumulated free Fe^2+^ then drives the Fenton reaction to generate highly toxic hydroxyl radicals, which triggers the chain reaction of membrane lipid peroxidation [65].

#### 5.2.2. The Integration of Exogenous PUFAs

Because native bacterial membranes inherently lack long-chain PUFAs, the incorporation of exogenous PUFAs (such as AA) significantly amplifies the membrane’s vulnerability to oxidative damage. This lethal lipid oxidation ultimately causes membrane perforation and the leakage of intracellular contents [66].

#### 5.2.3. The Collapse of the Bacterial Antioxidant System

Bacteria typically maintain a robust, multifaceted antioxidant defense network primarily relying on GSH, other low-molecular-weight thiols, and antioxidant enzymes. Ferroptosis-inducing treatments disrupt this system by directly depleting GSH and inhibiting key enzymes like catalase, thus stripping bacteria of their capacity to clear lethal lipid peroxides [67].

**Figure 3:**
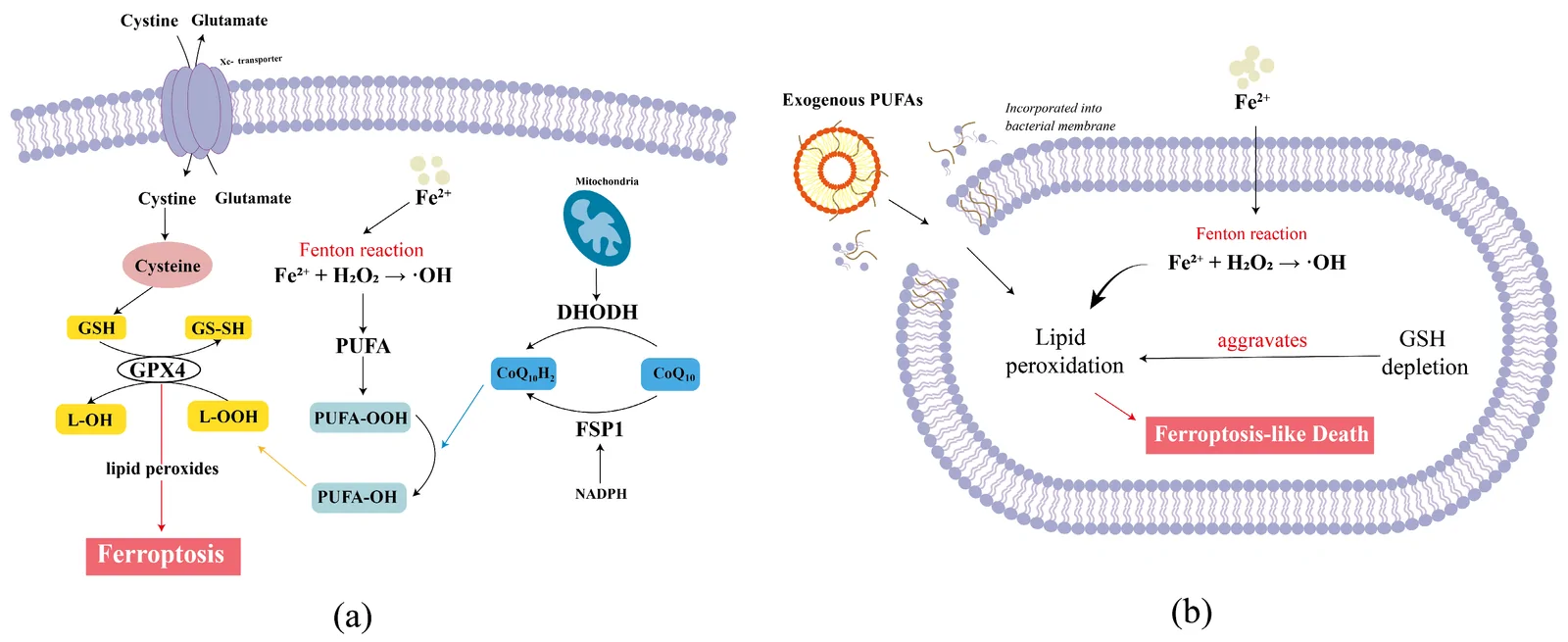
Mechanism of ferroptosis (**a**) and bacterial ferroptosis-like death (**b**).

The mechanism of ferroptosis and bacterial ferroptosis-like death is illustrated in Figure 3.

The bacterial ferroptosis-like death attracts the interest from therapists. For example, recently, pharmacological research has demonstrated that an Fe_3_O_4_-based ferroptosis inducer has been developed to induce ferroptosis in MRSA; hence disrupting bacterial biofilms and effectively treating acute MRSA pneumonia [68]. This finding confirms the feasibility of ferroptosis as an antibacterial strategy. Therefore, bacterial ferroptosis-like death can serve as a standalone therapy to induce bacterial death via its intrinsic mechanisms. Meanwhile, it also holds immense value as an adjuvant to conventional antibiotics. By depleting intracellular antioxidants (such as GSH) and increasing membrane permeability, this approach may effectively resensitize multidrug-resistant pathogens to traditional drugs.

Distinguishing bacterial ferroptosis-like death from non-specific oxidative killing requires more than relying on general ROS indicators; it demands a standardized set of experimental criteria. First, bacterial lethality must be primarily driven by envelope lipid peroxidation (LPO), quantifiable via lipophilic probes or malondialdehyde (MDA) assays. Crucially, specific rescue assays must be performed, showing that bactericidal efficacy is significantly attenuated by iron chelators (e.g., deferoxamine) or lipid radical scavengers (e.g., liproxstatin-1), rather than merely by general ROS scavengers [69].

Notably, the mechanisms driving bacterial ferroptosis-like death are distinct from classical bacterial drug resistance pathways, including (A) inactivating antibiotics through enzyme modification, (B) expressing of efflux pumps, (C) altering the molecular targets of antibiotics so that they cannot interact with antibiotics and (D) changing cell permeability to prevent antibiotics from entering cells. This unique characteristic suggests that inducing bacterial ferroptosis-like death strategies stand a good chance to possess the potential to overcome AMR, thus offering a promising and innovative approach to combating increasingly severe multidrug-resistant infections.

Based on the reliable research foundation, we hypothesized that ferroptosis-like death holds significant potential for application in the field of antibacterial therapy, especially in the treatment of drug-resistant bacterial infections caused by AMR.

## 6. Clinical Potential of Bacterial Ferroptosis-like Death-Inducing Nanomedicine

### 6.1. Bacterial Ferroptosis-like Death Inducers

To execute bacterial ferroptosis-like death, bacterial ferroptosis-like death inducers should be administrated. Commonly explored bacterial ferroptosis-like death inducers can be broadly classified into three categories based on their mechanisms of action:

#### 6.1.1. Iron-Based Nanomaterials and Nanozymes

These materials directly deliver exogenous ferrous ions and exhibit peroxidase-like activity. The agents catalyze the Fenton-mediated conversion of intracellular H_2_O_2_ into hydroxyl radicals (•OH), which subsequently trigger lipid peroxidation and membrane disruption in bacteria. A representative example is the Fe_3_O_4_-based ferroptosis inducer, which exploits this mechanism to exert potent bactericidal effects [68]. Furthermore, certain silver-magnetic nanomaterials possess excellent antioxidant capacities, which can drive the recycling of Fe^3+^ to Fe^2+^ within bacteria, thus accelerating the Fenton reaction to trigger lethal lipid peroxidation [70].

#### 6.1.2. Photodynamic Therapy or Sonodynamic Therapy Sensitizers Combined with Iron

Under light or ultrasound stimulation, photodynamic or sonodynamic sensitizers generate abundant ROS. When combined with exogenous iron supplementation or the disruption of bacterial iron storage proteins, this strategy rapidly triggers a lipid peroxidation storm akin to ferroptosis [71].

#### 6.1.3. Bacterial Metabolic Inhibitors

These agents suppress the synthesis of GSH or similar thiols within bacteria, thus weakening their antioxidant defenses. Coupled with environmental iron ions, they successfully induce ferroptosis-like death [72]. For instance, iron disulfide (FeS_2_) can directly oxidize and deplete GSH, fatally disrupting the bacterial GSH/oxidized glutathione (GSSG) ratio [73].

### 6.2. The Limitations of Inducers

Despite the promising translational potential of bacterial ferroptosis-like death as a novel antibacterial strategy to combat antimicrobial resistance stated previously, there are still two critical and significant limitations associated with current ferroptosis-like death inducers systems: poor aqueous solubility and insufficient targeting specificity, which severely hinder its clinical application, especially in antibacterial therapy.

For one thing, most of the identified or developed bacterial ferroptosis-like death inducers are small molecules. A predominant drawback of these small-molecule inducers is their poor water solubility, which leads to low systemic bioavailability, as most inducers fail to reach effective therapeutic concentrations at the infection site or within target bacterial cells [74].

For another thing, the current generation of bacterial ferroptosis-like death inducers universally lack satisfactory tissue specificity and precise targeting ability. Most unmodified bacterial ferroptosis-like death inducers distribute nonspecifically throughout the body after administration, may lead to ferroptosis in normal tissues (especially the liver, kidneys, and brain) [75].

### 6.3. The Use and Potential of Nanomedicine in Bacterial Ferroptosis-like Death

Nanocarriers are drug delivery systems engineered through nanotechnology [76], and they mainly include liposomes [77], vesicles [78], lipid nanoparticles (LNPs) [79], micelles [80], metallic nanoparticles [81], and so on. Nanocarriers offer significant advantages, including enhancing drug solubility and bioavailability [82], protecting therapeutic agents while prolonging their duration of action, and achieving precise targeted delivery. Most importantly, nanoparticle-based formulation strategies are very likely to address the limitations of bacterial ferroptosis-like death inducers.

Bacterial ferroptosis-like death inducers can be loaded onto nanocarriers to improve solubility and biocompatibility [83]. Take polymer micelles for instance: these colloidal aggregates form through the self-assembly of amphiphilic polymers, distinguished by their small size and superior solubility [84], can serve as effective carriers to encapsulate ferroptosis-inducing agents, therefore prolonging blood circulation time. When inducer self-assembles into micelles through the mixed amphiphilic polymer, its water solubility increases by more than 50 times, effectively improving the pharmacokinetic behavior in the body and prolonging the blood circulation duration [85].

Nanoparticle formulations mainly achieve precise drug delivery through three types of targeting mechanisms. The first is passive targeting, where the carrier does not require additional functionalization and relies on the nanoscale characteristics to achieve targeted enrichment at the target site through non-specific endocytosis of cells [86]; the second is active targeting, where specific targeting ligands are modified on the surface of the nanoparticle carrier, enabling precise recognition and binding to the high-expression receptors on the surface of target cells, and achieving targeted delivery through receptor-mediated endocytosis, with significant enhancement in targeting specificity [87]. For instance, surface-modified magnetic nanoparticles (MNP) with functional chemical groups can selectively interact with bacterial components and target bacteria [88]. In a biomimetic approach, nanocages composed of mesoporous silica nanoparticles, designated as Fe-G@MM, are cloaked in macrophage-like membranes. These carriers effectively target bacteria such as MRSA by generating ROS via the catalytic activity of iron ions and glucose oxidase loaded within the mesoporous silica nanoparticles (MSNs) [69]. The third aspect is environmental response targeting. By leveraging the physical and chemical differences between the lesion microenvironment and normal tissues, and through the design of pH-sensitive, redox-responsive, and enzyme-responsive structures, the carrier can specifically disintegrate in the lesion area and achieve controlled release of the drug [89]. In the context of bacteria-targeted modifications, a compelling example comes from the sonotherapeutic nano-ferrimycin. This system employs pH-responsive assembly to facilitate efficient drug internalization into bacteria. Its functionality extends beyond delivery: the intrinsic multimodal imaging signals (fluorescence, photoacoustic, and magnetic resonance imaging (MRI)) enable precise localization of infection foci [71].

In summary, passive accumulation at the infection site, biomimetic or receptor-mediated active targeting, and lesion-specific stimuli-responsive controlled release collectively establish a triple-layered selectivity system. This system spatially and temporally confines the ferroptosis-like antibacterial action, while concurrently minimizing off-target damage to host cells.

Currently, the documented research on ferroptosis-inducing nanomedicine in antibacterial therapy are summarized as follows in Table 2:

## 7. Liposomes Are Promising Nanocarriers for Bacterial Ferroptosis-like Death

In nanomedicine, there are two main components: drugs and nanocarriers. For the drugs, it has been confirmed that they are bacterial ferroptosis-like death inducers, such as Fe_3_O_4_, FeS_2_, etc. With respect to the nanocarriers, as mentioned earlier, they include liposomes, vesicles, LNPs, micelles, dendrimers, metallic nanoparticles, etc. Among them, we believe that liposomes are the most promising.

### 7.1. The Structural and Functional Benefits of Liposomes

Liposomes are vesicular structures formed by phospholipid bilayers encapsulating drugs and are widely known for their excellent biocompatibility and biodegradability [91]. As a drug delivery system, liposomes can encapsulate bacterial ferroptosis-like death inducers within their lipid bilayers and significantly enhance their aqueous solubility and systemic bioavailability [92]. Furthermore, the surface of liposomes can be chemically functionalized with specific ligands or functional groups to achieve precise bacterial targeting. This selective delivery approach minimizes off-target effects and optimizes drug selectivity and efficacy [93]. Critically, liposomes can solve the core challenges of poor solubility, low bioavailability and poor targeting of ferroptosis inducers.

In addition, liposomes offer several distinct advantages in drug delivery: Liposomes are compatible with biodegradable and non-toxic materials, reducing adverse effects on the human body and lowering the risk of long-term accumulation [94], avoiding the organ deposition and sustained off-target oxidative stress commonly associated with inorganic nanocarriers. Liposomes can also prolong the blood circulation time of drugs, which is beneficial for the retention and accumulation of liposome-loaded drugs at the diseased site. Meanwhile, encapsulation within the lipid bilayer protects free drugs from enzymatic degradation, chemical inactivation, and rapid clearance by the reticuloendothelial system; this shielding effect not only prevents premature metabolism before target tissue arrival but also minimizes toxicity to healthy tissues during circulation [95]. Collectively, the biodegradable composition, prolonged circulation and cargo-protective bilayer form a synergistic safety and delivery system, which is suitable for targeted anti-infective treatments based on bacterial ferroptosis-like death.

### 7.2. The Clinical Transformation History of Liposomes

Beyond their structural and functional benefits, liposomes possess a well-documented clinical translation [96]. Various marketed liposomal formulations are summarized in Table 3. Their versatility allows for the encapsulation of diverse cargoes, ranging from small-molecule antibiotics to biological components. In the future, as more diverse types of bacterial ferroptosis-like death inducers (such as protein-based or gene-based agents) are discovered. This broad applicability of liposomes could serve as a promising delivery platform.

However, while liposomes provide an advantageous starting point, the development of bacterial ferroptosis-inducing liposomes is still in its infancy and relies primarily on limited preclinical evidence. Notably, although liposomal nanomedicine have achieved clinical successes in cancer therapy, fungal diseases, and vaccines [92], these conventional formulations cannot be directly translated into antibacterial ferroptosis-like therapies. Structurally, the structural barriers are different, due to the presence of peptidoglycan layers and biofilms, commercially available liposomes often fail to penetrate the rigid bacterial cell walls and outer membranes [97]. Mechanistically, triggering ferroptosis-like death in bacteria mechanistically requires highly specific lipid compositions (such as specific PUFAs susceptible to peroxidation) that are not typically included in anti-cancer or vaccine liposomes [66].

## 8. Strategic Development of Liposome-Mediated Bacterial Ferroptosis-like Death for AMR Control

### 8.1. Mechanistic Advantages of Liposomes Against AMR

The escalating crisis of AMR is primarily driven by bacterial defense mechanisms, Liposomes offer distinct mechanistic advantages to circumvent these sophisticated defenses. As shown below:

#### 8.1.1. Bypassing Efflux Pumps and Membrane Impermeability

While free drugs rely on specific porins for intracellular entry, these channels are frequently down-regulated or mutated in resistant bacterial strains. To overcome this limitation, properly engineered liposomes (such as cationic or fusogenic liposomes) can directly interact and fuse with the bacterial envelope. This membrane fusion mechanism directly releases the encapsulated therapeutic agents into the bacterial cytoplasm, effectively bypassing porin-dependent entry channels and creating transient high intracellular drug concentrations that overwhelm bacterial efflux pumps [97].

Additionally, the lipid bilayer acts as a physical barrier, protecting the drug from degradation by bacterial enzymes. By masking the drug until it reaches the intracellular target, liposomes restore the efficacy of conventional antibiotics that would otherwise be neutralized [98].

#### 8.1.2. Disrupting Biofilm Microenvironments

Bacterial biofilms pose a formidable structural and functional barrier, conferring up to 1000-fold higher resistance than planktonic bacteria. Liposomes can be functionalized with specific surface charges, polymers, or targeting ligands to penetrate the EPS matrix. Furthermore, they can facilitate the co-delivery of anti-biofilm agents and antibiotics, dismantling the biofilm architecture while simultaneously eradicating the embedded persister cells [99].

#### 8.1.3. Potentiating Bacterial Ferroptosis-like Death

Most notably, in the emerging context of bacterial ferroptosis-like therapy, liposomes transcend their role as mere passive carriers. By incorporating specific PUFAs into their lipid bilayers, these nanovesicles may provide an abundant supply of peroxidizable lipid substrates. Upon entering the bacteria, these exogenous lipids integrate into the bacterial membranes and synergize with intracellular iron and ROS. This mechanism actively fuels catastrophic lipid peroxidation cascades, making the liposomes active drivers of bacterial ferroptosis-like death [100].

### 8.2. Current Challenges and Proposed Solutions

While integrating ferroptosis-inducing mechanisms into liposome systems provides a paradigm-shifting approach against bacterial infections, translating this concept into clinical practice requires overcoming barriers related to in vivo safety, delivery efficiency, and bacterial biofilm. The relevant content has been summarized in Table 4.

#### 8.2.1. Selectivity and Biosafety

The most critical bottleneck in developing antibacterial ferroptosis-like therapies is ensuring biosafety. Because canonical ferroptosis is a highly destructive mechanism in mammalian cells, non-specific distribution of these liposomes can cause severe host cytotoxicity. Premature release of PUFAs and iron modulators in the bloodstream can trigger systemic lipid peroxidation damage and massive off-target oxidative stress. Furthermore, excessive iron accumulation can lead to iron overload, provoking severe systemic inflammation. Given the reticuloendothelial system’s natural tendency to clear liposomes, significant accumulation in the liver and kidneys often results in unpredictable hepatotoxicity and nephrotoxicity [101].

Proposed Solution: To address this, liposome should be engineered with advanced bacterial-targeting and stimuli-responsive mechanisms. Designing smart liposome that degrade and release their payloads exclusively in response to the unique bacterial microenvironment (such as localized acidic pH, specific bacterial lipases, or elevated H_2_O_2_ levels) can confine the Fenton reaction strictly to the infection site, thus maximizing host biosafety.

#### 8.2.2. Efficiency and Stability of Lipid Delivery

Bacterial ferroptosis relies on the integration of exogenous PUFAs into the bacterial membrane to create a peroxidizable substrate. However, free long-chain PUFAs are chemically unstable and highly susceptible to premature autoxidation or protein binding during blood circulation, resulting in poor delivery efficiency [102].

Proposed Solution: Future research should focus on advanced encapsulation technologies to protect these vulnerable lipids. Strategies such as utilizing biomimetic cell membrane coatings, engineering highly stable liposomal formulations, or developing PUFA-conjugated prodrugs can effectively shield the lipids from premature degradation, ensuring their efficient insertion into bacterial membranes upon arrival [100,102].

#### 8.2.3. Biofilm Barrier

Chronic infections are frequently protected by biofilms, presenting a massive physical and biological barrier. Commercially available liposomes face restricted nanoparticle penetration due to the dense matrix of EPS, which acts as a molecular sieve and electrostatic trap. Furthermore, deep within the biofilm reside dormant cells that are metabolically inactive, rendering them highly tolerant to traditional antibiotics. This complex microenvironment engenders profound biofilm-specific resistance [103].

Proposed Solution: Overcoming this barrier requires dynamic liposomes designs. Developing size-switchable or charge-reversible liposomes can help the formulation navigate through the complex EPS pores. Furthermore, co-delivering biofilm-dispersing enzymes or coupling the therapy with physical disruption (such as photothermal or ultrasound stimulation) can effectively dismantle the EPS matrix, exposing deeply embedded bacteria to the ferroptosis nanomedicine [104].

## 9. Summary and Prospect

In summary, bacterial ferroptosis-like death, a novel iron-dependent cell death modality driven by lipid peroxidation, has emerged as a promising non-antibiotic strategy. While bacterial ferroptosis-like death inducer show great potential, their clinical application is hindered by poor solubility and selectivity. Nanomedicine, particularly liposomal delivery systems, offer a solution by enhancing drug stability, prolonging circulation time, and enabling targeted delivery to infection sites. The successful clinical approval of various liposomes formulations further underscores the translational viability of this approach.

Despite the promising progress, the following three issues remain to be addressed to facilitate the transition of ferroptosis-based liposomes from bench to bedside:

The efficacy of liposome-mediated bacterial ferroptosis-like death appears to be closely associated with the cell envelope architecture of the target bacteria. In Gram-negative bacteria, the lipid-rich outer membrane and LPS provide abundant substrates for rapid LPO, though they severely restrict the initial entry of liposomes. Conversely, in Gram-positive bacteria, liposomes need overcome the size-exclusion barrier of the thick hydrophilic peptidoglycan layer to reach the single underlying plasma membrane, where localized Fenton reactions and LPO can be lethally executed. These differences may affect the delivery of liposomes and the ultimate efficacy of ferroptosis.

Although liposome-mediated bacterial ferroptosis-like death has demonstrated antibacterial efficacy, there still exist critical issues for future research: under ferroptosis-like pressure, bacteria may develop adaptive strategies to ensure survival. Future studies should investigate whether bacteria can evade lethality through (i) antioxidant defenses, such as the compensatory upregulation of lipid hydroperoxidase or ROS-scavenging enzymes; (ii) iron regulation, by downregulating iron import receptors or overexpressing storage proteins like bacterioferritin to prevent intracellular iron overload; (iii) lipid remodeling, via altering the saturation degree of membrane fatty acids to reduce the oxidizable lipid pool; (iv) metal efflux, by hyperactivating multidrug efflux pumps to expel metal ions; or (v) enhanced biofilm formation, utilizing the dense EPS as a sacrificial shield. Elucidating these adaptive responses will be paramount for the rational design of next-generation, resistance-proof nanomedicine.

Translating ferroptosis-like liposomes still face manufacturing and regulatory hurdles. Successful industrial scale-up requires maintaining strict batch-to-batch reproducibility, particularly regarding particle size consistency and high encapsulation efficiency. Furthermore, guaranteeing long-term storage stability is a major challenge; developers must carefully optimize the lipid composition to prevent the premature leakage of iron-based payloads and the autoxidation of vulnerable PUFAs over time. From a regulatory standpoint, navigating complex safety guidelines is equally critical. This involves developing non-destructive sterilization techniques that do not compromise the integrity of heat-sensitive lipids, as well as enforcing rigorous endotoxin control to prevent severe immune reactions during intravenous administration.

We envision that, propelled by continuous progress in nanocarrier engineering and deepening cross-disciplinary convergence, liposome-mediated bacterial ferroptosis-like death hold the promise to emerge as a potent weapon against severe bacterial infections.

## Figures and Tables

**Table 1:** Common bacterial infections and their therapeutic drugs.

Category	Representative Drug	Mechanism	Indication	Reference
Penicillins	Amoxicillin	Binds to Penicillin-Binding Proteins (PBPs) to block peptidoglycan cross-linking	Skin infections or chest infections	[10]
Cephalosporins	Cefuroxime	Binds to PBPs to inhibit cell wall synthesis	Community-Acquired Pneumonia and meningitis	[11]
Carbapenems	Meropenem	Binds to PBPs with high affinity to inhibit cell wall synthesis	Bacterial meningitis	[12]
Monobactams	Aztreonam	Specifically binds to PBPs to inhibit cell wall synthesis in Gram-negative bacteria	Lung infection	[13]
Glycopeptide	Vancomycin	Binds to D-alanyl-D-alanine termini, physically blocking peptidoglycan elongation	Multidrug-resistant Gram-positive infections	[14]
Lipopeptides	Daptomycin	Inserts into the bacterial cell membrane, causing rapid depolarization and ion leakage	Bloodstream infections, pneumonia and urinary tract infections	[15]
Fosfomycins	Fosfomycin	Inhibits the MurA enzyme, blocking the very first step of peptidoglycan synthesis	Active against both Gram-positive and Gram-negative Multidrug-Resistant (MDR) and eXtensively Drug-Resistant (XDR) bacteria	[16]
Aminoglycosides	Gentamicin	Binds to the 30S ribosomal subunit, causing messenger ribonucleic acid (mRNA) misreading and halting synthesis	Gram-Negative pathogens infections	[17]
Lincosamides	Clindamycin	Binds to the 50S ribosomal subunit to inhibit peptide chain elongation	Anaerobic infections	[18]
Macrolides	Azithromycin	Binds to the 50S subunit, blocking the exit tunnel of growing peptide chains	Community-acquired pneumonia	[19]
Tetracyclines	Doxycycline	Binds to the 30S subunit, preventing the attachment of aminoacyl-tRNA	Acne and Rosacea	[20]
Quinolones	Ciprofloxacin	Inhibits deoxyribonucleic acid (DNA) gyrase and topoisomerase IV, preventing bacterial DNA replication	Respiratory and urinary tract infections	[21]
Rifamycins	Rifampicin	Binds to ribonucleic acid (RNA) polymerase, blocking the initiation of RNA transcription	Tuberculosis	[22]
Sulfonamides	Sulfamethoxazole	Competes with para-aminobenzoic acid (PABA) to inhibit dihydropteroate synthase, blocking folate synthesis	Streptococcal infection	[23]
Polypeptides	Polymyxin B	Binds to lipopolysaccharides (LPS), acting as a detergent to disrupt Gram-negative cell membranes	Carbapenem-resistant Gram-negative bacteria (CRGNB) infections	[24]

**Table 2:** Antibacterial application of ferroptosis-like nanoformulations.

Formulation	Infectionand Bacterial Strain	Animal Species and Infection Model	Route of Administration	Mechanism	Dose	Antibacterial Results	Security Data	Reference
Sonotherapeutic nano-ferrimycin	Pyomyositis; MDR *Pseudomonas aeruginosa *(PA)	Male Balb/c mice (~6 weeks and 20 g); MDR PA Induces Myositis in Mice	Local injection combined with ultrasound activation	Iron overload-induced bacterial ferroptosis-like killing	1.5 mg⋅kg^−1^ (calculated as sonosensitizer P18)	Near-complete pathogen eradication	The hemolysis rate was only 1.53% at a therapeutic concentration of 20 μM	[71]
Iron-loaded lipid nanoparticles (Fe-LNPs)	Skin infections; Staphylococcus aureus	Balb/c mice; A mouse model of Staphylococcus aureus skin wound infection	Topical application to the wound site	Lipid nanoparticles have high compatibility with bacterial cell membranes and are easily internalized by bacteria, delivering a large amount of ferric ammonium citrate (FAC) into the cells and inducing ferroptosis-like death	Low dose: 2 mM Fe-LNPs; High dose: 4 mM Fe-LNPs;	Wound bacterial clearance rate: 100% in the 4 mM group	The body weight of the 9-day cycle mice was normal, and there was no obvious irritation or aggravation of redness and swelling on the wound surface	[90]
Nanocages composed of mesoporous silica nanoparticles (Fe-G@MM)	Acute bacterial pneumonia and acute osteomyelitis; MRSA, PA	Balb/c mice; MRSA acute bacterial pneumonia mouse model and MRSA acute osteomyelitis mouse model	Tail vein injection treatment or local administration	Fe-G@MM can induce intracellular iron overload, GSH depletion, and lipid peroxidation, causing bacterial membrane breakdown via ferroptosis.	100 μL, preparation concentration 39.1 μg/mL (calculated as K_2_FeO_4_)	Lung injury was significantly lower than that in other groups. Effectively inhibit bacterial invasion and related bone damage	All treatment groups exhibited hemolytic activity below 5%	[69]

**Table 3:** Liposome products on the market.

No.	Type	Approval Year	Formulation & Administration Route	Product Name	Active Ingredient	Indications	Current Market Status
1	Lipid Complex	1995	Lipid suspension for IV infusion	Abelcet^®^	Amphotericin B	Invasive severe fungal infections	On Market
2	Conventional Liposome	1995	Sterile liposomal suspension for IV infusion	Doxil^®^/Caelyx^®^	Doxorubicin	Ovarian cancer and KS	On Market
3	Lipid Complex	1996	Lyophilized lipid suspension for IV infusion	Amphotec^®^	Amphotericin B	Severe fungal infections	Discontinued
4	Conventional Liposome	1996	Liposomal suspension for IV infusion	DaunoXome^®^	Daunorubicin	KS infected with HIV	Discontinued
5	Conventional Liposome (Vaccine liposome)	1997	Lipid adjuvant suspension for IM injection	Inflexal^®^ V	Inactivated hemagglutinin of influenza virus strains A and B	Influenza	Discontinued
6	Conventional Liposome	1997	Lyophilized liposomal powder for IV infusion	AmBisome^®^	Amphotericin B	Fungal infections	On Market
7	Conventional Liposome	1999	Liposomal suspension for intrathecal injection	Depocyt^®^	Cytarabine	Neoplastic meningitis	Discontinued
8	Conventional Liposome (Vaccine adjuvant liposome)	1999	Lipid suspension for SC injection	Epaxal^®^	Inactivated hepatitis A virus (strain RG-SB)	Hepatitis A	Discontinued
9	Conventional Liposome	2000	Lyophilized liposomal powder for IV infusion	Visudyne^®^	Verteporfin	Choroidal neovascularization	On Market
10	Conventional Liposome	2000	Three-component matched liposome for IV infusion	Myocet^®^	Doxorubicin	Combination therapy with cyclophosphamide in metastatic breast cancer	On Market (EU only)
11	Conventional Liposome	2003	Lyophilized liposomal powder for IV infusion	Lipusu^®^	Paclitaxel	Ovarian cancer	On Market (China only)
12	Conventional Liposome	2004	Liposomal suspension for epidural injection	DepoDur™	Morphine Sulfate	Pain management	On Market
13	Conventional Liposome	2009	Lyophilized liposomal powder for intra-articular injection	Mepact^®^	Mifamurtide	Non-metastatic osteosarcoma	On Market (EU orphan drug)
14	Conventional Liposome	2011	Liposomal suspension for surgical local infiltration	Exparel^®^	Bupivacaine	Pain management	On Market
15	Conventional Liposome	2012	Liposomal suspension for IV infusion	Marqibo^®^	Vincristine	ALL	Discontinued
16	Conventional Liposome	2015	Liposomal suspension for IV infusion	Onivyde™	Irinotecan	Metastatic pancreatic cancer	On Market
17	Conventional Liposome	2017	Liposomal suspension for IV infusion	Vyxeos^®^	Daunorubicin and Cytarabine	AML-MRC and AML	On Market
18	Conventional Liposome (Recombinant vaccine liposome)	2017	Lipid suspension for IM injection	Shingrix^®^	Recombinant VZV glycoprotein E	Against shingles and post-herpetic neuralgia	On Market
19	Conventional Liposome	2018	Nebulized liposomal inhalation suspension	Arikayce^®^ Kit	Amikacin	NTM lung disease caused by M. avium complex	On Market

**Table 4:** Current challenges and possible solutions.

Current Challenges	Specific Issues	Solutions
Selectivity & Biosafety	Potential host cytotoxicity. Systemic lipid peroxidation damage and off-target oxidative stress. Iron overload causing severe systemic inflammation. Hepatotoxicity and nephrotoxicity due to non-specific organ accumulation.	Engineering smart liposomes that respond to the local bacterial microenvironment; Employing surface functionalization strategies.
Efficiency and Stability of Lipid Delivery	Premature auto-oxidation of highly reactive PUFAs in the bloodstream. Low membrane fusogenicity of conventional liposomes.	Designing highly stable liposomal or exosome-mimetic delivery platforms; Employing prodrug strategies.
Biofilm Barriers	Restricted liposomes penetration due to the dense EPS matrix. Presence of metabolically inactive dormant cells.	Developing size-switchable or charge-reversible liposomes; Integrating photothermal therapy or ultrasound-mediated penetration technologies for biofilm disruption.

## Data Availability

No new data were created or analyzed in this study. Data sharing is not applicable to this article.

## Abbreviations

The following abbreviations are used in this manuscript:

AA	arachidonic acid
AdA	adrenic acid
AMPs	Antimicrobial peptides
AMR	antimicrobial resistance
CoQ_10_	coenzyme Q10
CoQ_10_H_2_	ubiquinol
CRGNB	Carbapenem-resistant Gram-negative bacteria
DHODH	dihydroorotate dehydrogenase
DNA	Deoxyribonucleic acid
EPIs	Efflux pump inhibitors
EPS	extracellular polysaccharides
FAC	ferric ammonium citrate
FSP1	Ferroptosis Suppressor Protein 1
GPX4	Glutathione peroxidase 4
GSH	glutathione
GSSG	oxidized glutathione
LNPs	lipid nanoparticles
LPO	lipid peroxidation
L-OH	non-toxic lipid alcohols
L-OOH	lipid hydroperoxides
LIP	labile iron pool
LPS	lipopolysaccharides
MDR	Multidrug-Resistant
MNP	magnetic nanoparticles
mRNA	messenger ribonucleic acid
MRI	magnetic resonance imaging
MRSA	Methicillin-resistant Staphylococcus aureus
MSNs	mesoporous silica nanoparticles
NADPH	nicotinamide adenine dinucleotide phosphate
PABA	Para-aminobenzoic Acid
PBPs	Penicillin-Binding Proteins
PUFAs	polyunsaturated fatty acids
PMF	proton motive force
ROS	reactive oxygen species
rRNA	ribosomal ribonucleic acid
WHO	The World Health Organization
XDR	eXtensively Drug-Resistant
